# Biomarkers of Methylmercury Exposure Immunotoxicity among Fish Consumers in Amazonian Brazil

**DOI:** 10.1289/ehp.1103741

**Published:** 2011-08-25

**Authors:** Jennifer F. Nyland, Myriam Fillion, Fernando Barbosa, Devon L. Shirley, Chiameka Chine, Melanie Lemire, Donna Mergler, Ellen K. Silbergeld

**Affiliations:** 1 Department of Pathology, Microbiology, and Immunology, School of Medicine, University of South Carolina, Columbia, South Carolina, USA; 2 Department of Environmental Health Sciences, Johns Hopkins Bloomberg School of Public Health, Baltimore, Maryland, USA; 3 Centre for Interdisciplinary Research on Biology, Health, Society, and Environment, Université du Québec à Montréal, Montréal, Québec, Canada; 4 Laboratório de Toxicologia e Essencialidade de Metais, Departamento de Análises Clínicas, Toxicológicas e Bromatológicas, Faculdade de Ciências Farmacêuticas de Ribeirão Preto, Universidade de São Paulo, Ribeirão Preto, Brazil

**Keywords:** autoantibodies, cytokine, immune response, mercury, selenium

## Abstract

Background: Mercury (Hg) is a ubiquitous environmental contaminant with neurodevelopmental and immune system effects. An informative biomarker of Hg-induced immunotoxicity could aid studies on the potential contribution to immune-related health effects.

Objectives: Our objectives were to test the hypothesis that methylmercury (MeHg) exposures affect levels of serum biomarkers and to examine interactions between Hg and selenium (Se) in terms of these responses.

Methods: This cross-sectional epidemiological study assessed adults living along the Tapajós River, a system long affected by MeHg. We measured antinuclear (ANA) and antinucleolar (ANoA) autoantibody levels and eight cytokines in serum samples (*n* = 232). Total Hg (including MeHg) and Se were measured in blood, plasma, hair, and urine.

Results: The median (range) total Hg concentrations were 14.1 μg/g (1.1–62.4), 53.5 μg/L (4.3–288.9), 8.8 μg/L (0.2–40), and 3.0 μg/L (0.2–16.1) for hair, blood, plasma, and urine, respectively. Elevated titers of ANA (but not ANoA) were positively associated with MeHg exposure (log-transformed, for blood and plasma), unadjusted [odds ratio (OR) = 2.6; 95% confidence interval (CI): 1.1, 6.2] and adjusted for sex and age (OR = 2.9; 95% CI: 1.1, 7.5). Proinflammatory [interleukin (IL)-6 and interferon (IFN)-©], anti-inflammatory (IL-4), and IL-17 cytokine levels were increased with MeHg exposure; however, in the subset of the population with elevated ANA, proinflammatory IL-1®, IL-6, IFN-©, and tumor necrosis factor (TNF)-〈 and anti-inflammatory (IL-4) cytokine levels were decreased with MeHg exposure. Although Se status was associated with MeHg level (correlation coefficient = 0.86; 95% CI: 0.29, 1.43), Se status was not associated with any changes in ANA and did not modify associations between Hg and ANA titers.

Conclusions: MeHg exposure was associated with an increased ANA and changes in serum cytokine profile. Moreover, alterations in serum cytokine profiles differed based on ANA response, suggesting a specific phenotype of MeHg susceptibility. Further research on the potential health implications of these observed immunological changes is warranted.

Mercury (Hg) is a global environmental contaminant and toxicant [Clarkson 1997; Gochfeld 2003; National Research Council (NRC) 2000]. Decades of releases of Hg (primarily from anthropogenic sources such as coal-fired power plants) have resulted in widespread contamination of ecosystems (NRC 2000). Much of airborne Hg pollution reaches aquatic systems, in which it is biomethylated to methylmercury (MeHg). In this form, it is readily taken up and bioaccumulated by fish through trophic webs, presenting the highest risks of exposure to top predators, including fish-eating birds, wild mammals, piscivorous fish (such as shark and tuna), and humans (NRC 2000). Fish consumption is an important source of omega-3 fatty acids and other components that promote prenatal development and protect against cardiovascular disease (von Schacky 2009). At the same time, certain fish species are a major source of exposure to MeHg (Groth 2010; Mahaffey et al. 2004), especially those from aquatic systems known to be contaminated by Hg inputs, such as the Amazon (Oliveira et al. 2010; Santos et al. 2000).

In the Amazonian region of Brazil, contamination of aquatic systems and subsequent increases in levels of MeHg in fish have been associated with the use of elemental Hg in gold mining as well as deforestation and erosion resulting in releases of inorganic Hg from soils (Guimaraes et al. 2000; Sampaio da Silva et al. 2009). Populations, including this cohort, have been reported previously to have elevated levels of Hg in hair and blood associated with rates of consuming MeHg-contaminated fish. Health effects have also been reported in these populations, including neurodevelopmental toxicity, cognitive deficits, reduced visual functions, motor function impairments, and alterations in cardiovascular function (Passos and Mergler 2008; Yokoo et al. 2003).

Although there have been many studies on neurotoxic effects of Hg exposures in Amazonia and elsewhere (Dolbec et al. 2001; Grandjean et al. 1999; Yokoo et al. 2003), recent experimental and epidemiological studies by us and others have suggested that these exposures, to both inorganic and MeHg, may affect the immune system (Alves et al. 2006; Gardner et al. 2010b; Silva et al. 2004). Previous studies have reported an association between Hg exposures in Brazil among gold miners and fish consumers and increased levels of antinuclear (ANA)/antinucleolar (ANoA) autoantibodies, interpreted as biomarkers of autoimmune dysfunction (Alves et al. 2006; Gardner et al. 2010b; Silva et al. 2004). However, these studies were conducted in persons with higher Hg exposures than those reported for the fish-consuming population (Fillion et al. 2006) examined in this manuscript and for gold miners in particular. Conversely, in a study of paired maternal–fetal blood samples (also from the Brazilian Amazon), no relationship was found between ANA/ANoA titer and MeHg level (Nyland et al. 2011). We hypothesize that this is because of the differential immune responses of the fetus and pregnant female. Our previous studies have been relatively small and included, as mentioned, exposures to both inorganic and MeHg. This project was undertaken to further our understanding of the immunotoxicology of MeHg in humans within a much larger population.

ANA and ANoA autoantibodies are self-reactive antibodies found in the serum; positive ANA titers are used as one of the panel of diagnostic criteria for clinical lupus (Tan et al. 1982). In studies of communities and populations exposed to either inorganic or MeHg in the Brazilian Amazon, we and others have reported a correlation with increased prevalence of elevated ANA/ANoA titers (Alves et al. 2006; Gardner et al. 2010b; Silva et al. 2004) and proinflammatory cytokines (Gardner et al. 2010b). Furthermore, we have recently shown that *in vitro* exposure of human peripheral blood mononuclear cells to inorganic Hg at physiologically relevant doses (< 200 nM) induces an unopposed inflammatory response (Gardner et al. 2009). Inflammatory cytokine production was consistently increased in a dose–response manner for proinflammatory cytokines interleukin (IL)-1® and tumor necrosis factor (TNF)-〈, with a concurrent decrease in anti-inflammatory cytokine production. Because systemic cytokine levels, as found in serum, can serve as measurable biomarkers of an inappropriate immune response, these results suggest that low exposures to inorganic or MeHg can affect cytokine-signaling pathways and potentially dysregulate the immune response.

In this study, we examined serum biomarkers in a cohort of fish consumers in Brazil to test associations between biomarkers of MeHg exposure and elevated ANA/ANoA and proinflammatory cytokines. We hypothesize that elevated MeHg exposure increases the likelihood for increases in ANA/ANoA as well as increased serum levels of proinflammatory cytokines. Whether these increases are sufficient to increase risks of clinical disease is not known; in this study we used these measurements as potential biomarkers of immune response to MeHg exposures. In addition, we considered the potential for interactions between MeHg exposure and selenium (Se) status, as there are reports in experimental and clinical studies that this element (Se) can affect immune function (Beck et al. 2003; Hoffmann and Berry 2008) and in epidemiological studies that Hg and Se can have opposing influences on certain diseases (Lemire et al. 2010). In populations from the Brazilian Amazon region, Se status ranges from normal to relatively high (Lemire et al. 2006, 2009).

## Methods

This work was conducted with approval from the institutional review boards of the University of South Carolina School of Medicine, the Johns Hopkins Bloomberg School of Public Health, the University of Quebec at Montreal, and the University of São Paulo–Ribeirão Preto.

*Study design.* The subjects in this cross-sectional epidemiological study were part of an ongoing larger project using an integrated approach to examine factors that modulate MeHg transmission through aquatic ecosystems, human exposures, and health effects in populations residing in the Lower Tapajós River Basin in the Brazilian Amazon (Lemire et al. 2006). This region has been affected by both deforestation and erosion as well as by a history of Hg use in small-scale gold extraction (Silbergeld et al. 2005). Over the past two decades, populations in this region have been studied from approximately 50 communities with diverse population sizes and time of residence and differences in diet as well as occupational histories involving use of Hg in gold mining, in addition to fishing and traditional agricultural practices (Passos and Mergler 2008).

*Study population.* Subject enrollment, assessment, and biological sample collections were completed during the summer of 2006 using a convenience sampling method (Fillion et al. 2006). The representativeness of the participating subjects to the mid-Tapajós communities was assessed by collecting information on the distribution of age and sex through door-to-door sociodemographic surveys in the communities. Several studies in this field and region, including this project, have used convenience sampling methods. In this setting, it is difficult to use randomized or population-based designs, given the lack of baseline information on many communities, the relatively small size of each community, and the barriers to participation such as lack of transportation. This latter barrier was reduced by providing transportation for the participants from their riverside villages to a central study site in the city of Itaituba.

A total of 448 persons participated in the parent study over two enrollment periods separated by 3 weeks in 2006. The following information was obtained by questionnaire [pilot tested and administered in Portuguese (Fillion et al. 2006)]: sociodemographics, health history, self-reported current health status, residence, occupational history, and diet (7-day recall food frequency questionnaire). MeHg exposure was identified by questionnaire responses and directly determined by measuring total Hg (including MeHg) in whole blood, plasma, hair, and urine; Se was measured in these same compartments. For these analyses, we used the samples from the first enrollment period: these samples were from 232 adults between 15 and 87 years of age (112 males and 120 females, evenly distributed by sex across the age range) from six villages. All participants provided informed consent. Details of the subject recruitment and demographic characteristics of the participants have been previously reported (Barbosa et al. 2009).

*Blood sample collection and metals analysis.* For each participant, an experienced Brazilian nurse collected an 8-mL blood sample in trace metal–free evacuated tubes (Vacutainer®), some containing heparin as an anticoagulant (depending on the sample needs). For plasma separation, blood samples were centrifuged (800 × *g* for 6 min). Plasma fractions were then aliquoted and immediately frozen at –20°C. Blood total Hg (including MeHg), blood Se, plasma total Hg, and plasma Se were determined by inductively coupled plasma mass spectrometry (ICP-MS; PerkinElmer DRC II; PerkinElmer, Wellesley, MA, USA) according to the method proposed by Batista et al. (2009) at the Laboratório de Toxicologia e Essencialidade de Metais. The detection limits are 0.08 and 0.10 µg/L for Hg in plasma and whole blood, respectively. For Se, the detection limits are 0.14 and 0.18 μg/L in plasma and whole blood, respectively. For these analyses, Hg speciation was not available.

*Hair sampling and determination of Hg and Se.* Hair samples were taken close to the scalp from the occipital area of the head. The lock of hair was stapled at the base and stored in identified plastic bags. For this analysis, the first centimeter of hair from the scalp was used. Total Hg concentrations were determined by cold vapor atomic absorption spectrometry according to the method described by Ebbestadt et al. (1975). Se was determined by ICP-MS according to the method of Rodrigues et al. (2008) at the Laboratório de Toxicologia e Essencialidade de Metais. The detection limit is 4.0 ng/g. For these analyses of hair Hg, speciation into inorganic, organic, and total Hg was performed.

*Urine sampling and determination of Hg and Se.* Urine samples were collected into washed polypropylene bottles and then transferred to tubes for transport; samples were frozen at –20°C until analysis. Urine Hg and Se were measured by ICP-MS according to the method of Goulle et al. (2005) at the Laboratório de Toxicologia e Essencialidade de Metais. The detection limit is 0.08 and 0.05 μg/L for Hg and Se, respectively.

*Quality control of the results.* Quality control of Hg and Se determination in blood, hair, and urine samples was guaranteed by analyzing standard reference materials produced by the National Institute of Standards and Technology (SRM 2670a low and high level) and International Atomic Energy Agency (CRM 85 and 86 human hair). In addition, various secondary reference materials provided by the Institut National de Santé Publique du Québec, Canada (external quality assessment scheme for trace elements in blood, serum, hair, and urine), and by the New York State Department of Health’s proficiency test program for trace elements in whole blood and serum were analyzed. We analyzed reference samples before and after analyzing 10 ordinary samples. All results were within the target values.

*Immunoglobulin and serum analyses.* Frozen serum samples were coded and sent to Johns Hopkins for immunological analyses under blinded conditions. Total serum ANA/ANoA was measured with indirect immunofluorescence microscopy using commercially available slides prepared from human epithelial cells (HEp-2) as substrate (INOVA Diagnostics, San Diego, CA, USA), as described previously (Burek and Rose 1995); these are the same methods applied in our studies of adults exposed to inorganic and MeHg (Gardner et al. 2010b; Nyland et al. 2011; Silva et al. 2004). Briefly, we tested serum samples in 2-fold serial dilutions starting at 1:10. The inverse of the highest dilution at which fluorescence could still be detected was defined as the titer. A negative result at the lowest dilution (1:10) was defined as a titer of zero.

*Cytokine measurement.* Sample selection. For these analyses, cytokines were measured in the entire cohort (*n* = 232) with ANA/ANoA titer and Hg level blinded. For nested statistical analyses, sample identity was unmasked after ANA/ANoA and cytokine analysis to allow division into four groups, dichotomized as either high or low Hg and either highly positive or negative for ANA/ANoA. Because samples were noted to be either positive for ANA alone or positive for both ANA and ANoA, the positive ANA/ANoA category (ANA/ANoA+) includes either only ANA positive or both ANA and ANoA positive. The negative ANA/ANoA category includes only those samples that were negative for both ANA and ANoA. The groups were as follows: *a*) high Hg and ANA/ANoA+; *b*) high Hg and ANA/ANoA negative; *c*) low Hg and ANA/ANoA+; and *d*) low Hg and ANA/ANoA negative. High Hg was defined as total blood Hg levels in the fourth quartile of Hg exposure (range 78.6–288.9 μg/L); low Hg was defined as total blood Hg levels in the first quartile (range 10.3–21.4 μg/L). ANA/ANoA negative samples were those with nondetectable antibody at a serum dilution of 1:10; ANA/ANoA+ samples were those with an ANA or ANoA titer ≥ 1:80. This group division resulted in a reduced number of samples for the nested analysis (*n* = 96). These four groups were established as in our previous studies of serum cytokines in Hg-exposed gold miners (Gardner et al. 2010b).

*Cytokine analysis.* An aliquot of each selected serum sample was analyzed for cytokine content using the BioPlex multiplex bead-based cytokine assay (Bio-Rad Laboratories, Hercules, CA) according to the manufacturer’s instructions. The following eight cytokines were measured: IL-1®, IL-1 receptor agonist (IL-1ra), IL-4, IL-6, IL-10, IL-17, interferon (IFN)-©, and TNF-〈. These cytokines were selected based on our studies of Hg exposures *in vitro* and *in vivo* (Gardner et al. 2009, 2010b). Briefly, color-coded fluorescent beads conjugated with antibodies specific for the cytokine of interest were incubated at room temperature with 50 μL diluted serum sample. After washing, the beads were incubated with biotinylated detection antibody. The beads were washed and treated with streptavidin-PE to detect the bound antibodies. The beads were then analyzed using the Bio-Plex Suspension Array system (Bio-Rad Laboratories), a flow-based instrument with the ability to detect the presence of the PE and the fluorescence range of each bead. Using information from a standard curve (created in triplicate) for each cytokine measured, it was possible to infer the concentration of each cytokine present in the sample. Cytokine measurements below the limit of detection as determined by the standard curve for each individual cytokine were assigned a value of the limit of detection/_√_^–^2 for statistical analysis and plotting.

*Statistical analyses.* Because Hg levels (blood, plasma, hair, and urine) are right-skewed in this as in most studies of Hg exposure, including the overall cohort from which this sample was drawn (Fillion et al. 2006) and our other studies (Crompton et al. 2002; Gardner et al. 2010b; Silva et al. 2004), we log-transformed these data for analysis. Se and cytokine levels were also right-skewed, and therefore, we also log-transformed that data for analysis. Evaluation of the association of Hg and/or Se levels with biomarkers of immune response was completed with linear regression modeling for continuous biomarkers of immune response (cytokines) or logistic regression analysis for categorical biomarkers (ANA/ANoA). Evaluation of the association of Hg levels with ANA titer was completed with logistic regression, using quartile of Hg exposure as a categorical variable. Log-transformed cytokine values were compared using one-way analysis of variance (ANOVA) followed by pairwise post-tests using the Bonferroni correction for the nested analysis. Models were adjusted for age and sex. Modification of associations between Hg and biomarkers of immune response by Se status was tested with addition of log-transformed Se level to the model tested. Where no change in significance was noted with this addition, we concluded that there was no modification of association by Se. All analyses were conducted in STATA (version 10_IC; StataCorp LP, College Station, TX). We considered *p* < 0.05 to be significant.

*Human subjects research approval.* The study was approved by the institutional review boards of the University of South Carolina School of Medicine, the Johns Hopkins Bloomberg School of Public Health Committee for Human Research, the University of Quebec at Montreal, and the University of São Paulo–Ribeirão Preto. All participants signed a consent form after it was read to them in Portuguese.

## Results

**Figure 1:**
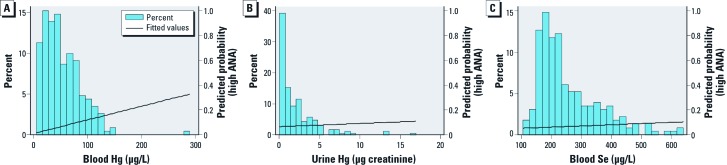
Hg and Se distribution in population. Total Hg levels (*A,B*) and total Se levels (*C*) were measured for each participant in (*A*,*C*) blood and (*B*) urine (creatinine-corrected values). Data are shown on the natural scale and are right-skewed as in previous studies of individuals from this study area. The predicted probability (line overlay) of high (titer > 1:80) ANA measures in serum is plotted as relates to (*A*) blood Hg (*p* = 0.031; OR = 2.6; 95% CI: 1.1, 6.2), (*B*) urine Hg (*p* = 0.70; OR = 1.2; 95% CI: 0.5, 3.1), and (*C*) blood Se (*p* = 0.57; OR = 2.5; 95% CI: 0.1, 62.8).

*Hg exposure and population characteristics.* The range of blood Hg levels in this population included the range of values reported in U.S. populations (Mahaffey et al. 2004), but the upper limit was substantially greater (Figure 1), and the geometric mean (42.5 μg/L) was significantly above that reported by Mahaffey et al. (U.S. geometric mean: 1.02 μg/L; nondetected = 0.14 μg/L; 95th percentile = 7.13 μg/L). This value also exceeds recommended exposure limits for pregnant women and children in the United States (NRC 2000). These elevated levels were likely due in large part to the rates of self-reported fish consumption, which were significantly higher than those for most of the U.S. population (Mahaffey et al. 2004), as well as the known contamination of fish from the river ecosystem, as described above. Fish consumption rates are known to be highly associated with biomarkers of Hg exposure in this population as well as in the United States, Canada, Sweden, and Brazil (Mahaffey et al. 2004; Mahaffey and Mergler 1998; Passos et al. 2007; Vahter et al. 2000), especially when the type of fish is taken into account (Abdelouahab et al. 2008). As in previous studies in this population (Lemire et al. 2009), we found a positive correlation between blood Hg and Se in individuals’ [correlation coefficient (*r*) = 0.1; 95% confidence interval (CI): 0.04, 0.17].

**Figure 2:**
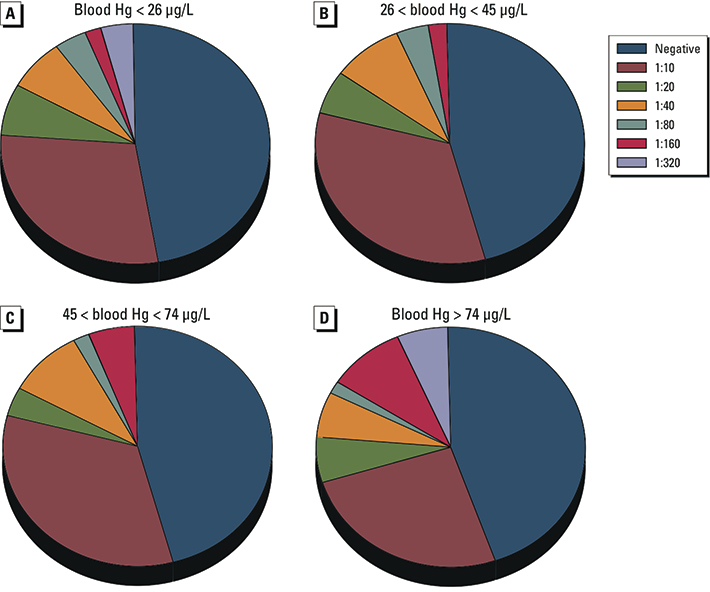
ANA titer by blood Hg quartile. Serum samples from the study population (*n *= 232) were serially diluted and assayed for ANA. Results show the proportion of the population for each blood Hg quartile that had detectable amounts of ANA at the titer noted, with negative indicating that no ANA was detectable at any dilution. Significance of associations between elevated titers of ANA and Hg exposure was evaluated with logistic regression, using quartile of Hg exposure as a categorical variable. Positive associations between percent positive for high ANA (defined as > 1:80) and elevated Hg exposure (by quartile) were found between the first and fourth Hg quartile (OR = 3.0; 95% CI: 0.99, 9.0).

*Elevated ANA titers were associated with log-transformed changes in Hg but not Se level.* ANA and ANoA were analyzed in serum samples from all participants. There was a significant positive association (Table 1) between high ANA titers (> 1:80, comparison group < 1:80) and blood [odds ratio (OR) = 2.6; 95% CI: 1.1, 6.2] and plasma (OR = 19.9; 95% CI: 2.4, 163.9) Hg levels (Figure 1A line overlay for blood), but not hair Hg (neither total, inorganic, or organic Hg) levels or urine Hg levels (Figure 1B line overlay) corrected by creatinine value. ORs were similar when adjusted for age and sex. Using a lower cutoff value to define elevated ANA titers (defined as > 1:40), there was a positive but nonsignificant association between antibody titers and blood Hg (OR = 2.1; 95% CI: 0.72, 5.9). Binning the data by Hg quartile (Figure 2) indicated a dose-related association between ANA and blood Hg (percent high ANA for quartile: Q1 = 8.6, Q2 = 5.2, Q3 = 12.1, and Q4 = 22.0) with differences between the first and fourth quartiles (OR = 3.0; 95% CI: 0.99, 9.0). None of the measures of Se (hair, blood, plasma, or urine) were significantly associated with ANA or ANoA titers, nor did Se levels modify significance of associations between Hg and elevated ANA (data not shown). Similarly, no association was found between ANoA and Hg or Se in blood, plasma, hair, or urine (data not shown).

**Table 1:** Associations between ANA and Hg level (OR and 95% CI for a log change in Hg exposure).

Biomarker	Blood Hg	Plasma Hg	Hair Hg	Urine Hg*a*
Unadjusted associations								
High ANA (≥ 1:80)		2.6 (1.1, 6.2)		19.9 (2.4, 163.9)		3.4 (0.6, 19.2)		1.2 (0.5, 3.1)
Mid-ANA (≥ 1:40)		1.3 (0.8, 2.2)		2.1 (0.8, 5.7)		1.6 (0.5, 4.9)		0.7 (0.4, 1.4)
Adjusted associations*b*								
High ANA (≥ 1:80)		2.8 (1.1, 7.5)		28.3 (2.9, 273.2)		3.9 (0.6, 24.3)		1.2 (0.5, 3.2)
Mid-ANA (≥ 1:40)		1.3 (0.8, 2.2)		2.1 (0.8, 5.6)		1.5 (0.5, 4.6)		0.7 (0.4, 1.4)
Values [OR (95% CI)] are reported for unadjusted and adjusted biomarkers. **a**Urine Hg corrected for creatinine content. **b**Adjusted for age and sex.								

*Both pro- and anti-inflammatory cytokines were increased with Hg exposure.* Eight cytokines were measured in the serum samples of the entire cohort. Regression analysis showed that levels of both pro- and anti-inflammatory cytokines were increased with increased Hg exposure (Table 2). There was a positive association between IL-4 levels and plasma (*r* = 0.5; 95% CI: 0.1, 0.9) and blood Hg (*r* = 0.5; 95% CI: 0.0, 1.0), but not hair or urine Hg. IL-6 serum levels were positively associated with blood (*r* = 0.6; 95% CI: 0.0, 1.1), plasma (*r* = 0.4; 95% CI: 0.0, 0.8), and hair (*r* = 0.5; 95% CI: 0.0, 1.0), but not urine Hg. Serum IL-17 levels were positively associated with blood (*r* = 0.2; 95% CI: 0.0, 0.5), plasma (*r* = 0.2; 95% CI: 0.0, 0.4), and hair (*r* = 0.2; 95% CI: 0.0, 0.4), but not urine Hg. There was a positive association between elevated IFN-© levels and plasma (*r* = 0.9; 95% CI: 0.2, 1.5) and blood (*r* = 0.8; 95% CI: –0.1, 1.6), but not hair or urine Hg. Associations were similar when adjusted for age and sex.

**Table 2:** Associations between serum cytokines and Hg level [*r *(95% CI)].

Cytokine	Blood Hg	Plasma Hg	Hair Hg	Urine Hg*a*
Unadjusted associations								
IL-1β		0.2 (–0.4, 0.7)		0.0 (–0.4, 0.4)		0.0 (–0.5, 0.5)		0.0 (–0.1, 0.1)
IL-1ra		0.0 (–0.5, 0.6)		–0.3 (–0.7, 0.1)		0.0 (–0.5, 0.6)		0.0 (–0.1, 0.0)
IL-4		0.5 (0.0, 1.0)		0.5 (0.1, 0.9)		0.3 (–0.2, 0.8)		0.0 (0.0, 0.1)
IL-6		0.6 (0.0, 1.1)		0.4 (0.0, 0.8)		0.5 (0.0, 1.0)		0.1 (0.0, 0.1)
IL-10		0.2 (–0.2, 0.5)		–0.1 (–0.3, 0.2)		0.1 (–0.3, 0.5)		0.0 (0.0, 0.1)
IL-17		0.2 (0.0, 0.5)		0.2 (0.0, 0.4)		0.2 (0.0, 0.4)		0.0 (0.0, 0.0)
IFN-γ		0.8 (–0.1, 1.6)		0.9 (0.2, 1.5)		0.5 (–0.3, 1.3)		0.1 (0.0, 0.2)
TNF-α		0.0 (–0.4, 0.4)		0.1 (–0.2, 0.4)		–0.1 (–0.5, 0.3)		0.0 (0.0, 0.1)
Adjusted associations*b*								
IL-1β		0.1 (–0.5, 0.7)		–0.1 (–0.5, 0.4)		–0.1 (–0.6, 0.5)		0.0 (–0.1, 0.1)
IL-1ra		0.1 (–0.5, 0.6)		–0.3 (–0.7, 0.1)		0.1 (–0.5, 0.6)		0.0 (–0.1, 0.1)
IL-4		0.4 (–0.1, 0.9)		0.5 (0.1, 0.9)		0.2 (–0.3, 0.7)		0.0 (0.0. 0.1)
IL-6		0.6 (0.0, 1.2)		0.4 (0.0, 0.9)		0.5 (0.0, 1.1)		0.1 (0.0, 0.1)
IL-10		0.1 (–0.3, 0.5)		–0.1 (–0.4, 0.2)		0.1 (–0.3, 0.5)		0.0 (0.0, 0.1)
IL-17		0.2 (–0.1, 0.4)		0.2 (0.0, 0.4)		0.1 (–0.1, 0.4)		0.0 (0.0, 0.0)
IFN-γ		0.7 (–0.2, 1.6)		0.8 (0.2, 1.5)		0.5 (–0.4, 1.3)		0.1 (0.0, 0.2)
TNF-α		0.0 (–0.4, 0.4)		0.1 (–0.2, 0.4)		–0.2 (–0.5, 0.2)		0.0 (0.0, 0.1)
Values are reported for unadjusted and adjusted biomarkers. **a**Urine Hg corrected for creatinine content. **b**Adjusted for age and sex.								

*Both pro- and anti-inflammatory cytokines were decreased with Hg exposure for ANA+ groups.* For the second part of the cytokine analyses, we carried out a nested analysis of the same cohort by dividing the serum samples into four groups based on ANA status and Hg exposure, as shown in Table 3. This is similar to the analytic strategy used in our study of miners and Hg exposure (Gardner et al. 2010b). ANOVA analysis showed that levels of the proinflammatory cytokines TNF-〈 (*p* < 0.05), IFN-© (*p* < 0.001), IL-6 (*p* = 0.01), and IL-1® (*p* = 0.05) were significantly associated with Hg/ANA status (Table 3). Conversely, levels of the anti-inflammatory cytokine IL-4 (*p* < 0.0001) were also associated with Hg/ANA status. All six of these cytokines, both pro- and anti-inflammatory, were decreased in the high Hg and ANA/ANoA+ group compared with the low Hg and ANA/ANoA negative group (Table 3).

**Table 3:** Serum cytokine measurements [pg/mL; median (interguartile range)] according to ANA status and Hg exposure.

Group*a*	*n*	Hg level [median (CI)]*b*	IL-1β *p* = 0.03	IL-4 *p* < 0.001	IL-6 *p* = 0.01	IFN-γ *p* < 0.001	TNF-α *p* = 0.004
High Hg/ANA+		9		103.1 (79.4, 122.1)		1.5 (1.5–2.1)		1.3 (1.0–1.8)		5.0 (3.5–7.2)		70.1 (50.5–90.4)		7.9 (6.7–15.2)
High Hg/ANA–		39		93.6 (84.6, 101.3)		0.7 (0.5–1.5)		0.2 (0.2–0.2)		1.4 (1.2–6.3)		1.3 (1.3–1.7)		4.6 (3.2–4.7)
Low Hg/ANA+		5		21.1 (16.8, 25.3)		2.2 (1.8–2.5)		2.2 (1.5–3.0)		5.8 (4.0–7.6)		71.2 (52.1–136.0)		12.0 (7.4–20.2)
Low Hg/ANA–		43		15.3 (13.1, 19.4)		0.7 (0.2–1.5)		0.2 (0.2–0.2)		1.2 (1.2–2.6)		1.3 (1.3–1.7)		4.6 (4.5–4.7)
ANOVA *p*-value.****a**High Hg grouping includes samples in the top (fourth) quartile; low Hg grouping includes samples in the bottom (first) quartile; ANA+ grouping includes samples with a titer > 1:80; ANA negative grouping includes samples with a titer < 1:10. **b**Hg levels are measured in blood (micrograms per liter).														

## Discussion

For this study, we performed an analysis of immune biomarkers in a population from Amazonian Brazil with a range of exposures to MeHg exclusively through fish consumption. We found that elevated MeHg exposures were associated with high titers of ANA, but not ANoA, in the serum. These positive associations were unaffected by adjustment for Se status, sex, or age.

These results are consistent with previous studies by us and others on the immunomodulatory effects of Hg on humans exposed to Hg in the Brazilian Amazon (Alves et al. 2006; Gardner et al. 2010b; Silva et al. 2004). These studies demonstrate a positive association between Hg exposure and elevated ANA levels in serum. In studies of fish consumers (Alves et al. 2006), elevated ANA levels were associated with fish consumption from regions known to contain MeHg-contaminated fish (Passos et al. 2008). Studies of *in vitro* human immune cell response to Hg have demonstrated that inorganic Hg at physiologically relevant low exposures induces a proinflammatory cytokine response that was not accompanied by an induced anti-inflammatory cytokine response (Gardner et al. 2009). Similar results were found for cytokine responses to ethyl Hg and MeHg *in vitro* (Gardner et al. 2010a). In this current study, individuals exposed to MeHg through fish consumption had higher serum concentrations of both proinflammatory and anti-inflammatory cytokines (Table 2). We hypothesize that *in vivo* exposures in this study, compared with *in vitro* exposures, and the form of Hg to which these individuals were primarily exposed (organic vs. inorganic) may have contributed to the differences in these findings and those of Gardner et al. (2009, 2010a).

Interestingly, when including positivity for ANA in the analysis [as in Gardner et al. (2010b)], individuals in this study with high Hg exposure and a high (> 1:80) positive ANA titer had lower pro- and anti-inflammatory cytokines than those individuals with low Hg exposure. IL-6 serum levels (Table 3) were also decreased in those individuals with high Hg exposure and high positive ANA titer compared with those with low Hg exposure and also ANA positive. These results are inconsistent with previous findings in susceptible murine models of Hg-induced lupus, in which elevated IL-6 has been found to be critical for the pathogenicity of the disease (Havarinasab et al. 2005, 2009). However, the results of this study are consistent with *in vitro* studies showing that Hg exposure reduces IL-6 production by various cell types, including human immune cells (Hemdan et al. 2007). Differences in routes of exposure, form of Hg, and level of exposure could explain the inconsistencies in these findings.

Hg has been hypothesized to interact with Se, an essential trace element necessary for many biological and enzymatic activities, although the molecular mechanisms of the interactions and their toxicity remain unclear (Khan and Wang 2009). Studies in this same population reported a positive relationship between blood levels of Hg and Se in individuals (as we have also reported here) (Lemire et al. 2006). We observed no significant associations between blood (or hair, plasma, or urine) Se levels and risk of elevated ANA/ANoA titer either on its own or as a modulator of the associations between MeHg and ANA/ANoA, suggesting that this relationship did not influence the immunotoxic effects of MeHg.

We found that elevated ANA, but not ANoA, titers are significantly associated with MeHg exposure, confirming our previous studies of individuals exposed occupationally to inorganic Hg (artisanal gold miners) or through fish consumption to MeHg (Gardner et al. 2010b; Silva et al. 2004). The similarities for these two studies are found when the total ANA/ANoA (as defined by individuals with either ANA or ANoA or both) titers are used; in the study by Silva et al. (2004), the percentage of the contaminated fish–consuming population (village of Jacareacanga) with elevated ANA (as opposed to ANoA) was much lower than that with elevated ANoA. Thus, when the total ANA/ANoA positive portion of the Jacareacanga population is used to analyze effects of MeHg exposure, the results are similar to those found here. We hypothesize that this difference in MeHg association with elevated ANoA between the study by Silva et al. (2004) and this study is due to the lower range of MeHg exposure in the Jacareacanga population.

Although ANA/ANoA is one of the biomarkers used to diagnose certain autoimmune diseases, the interpretation of changed serum levels in the absence of other health information is limited. Based on the information in this study, it is not appropriate to infer any increased risk of a clinical or disease outcome. Rather, we consider these data indicative of an immunotoxic or immunomodulatory effect of MeHg exposures over the range found in this population, which may be relevant to increased risks of autoimmune dysfunction in the broad sense. The general finding that elevated MeHg exposure was associated with increased levels of serum cytokines, both pro- and anti-inflammatory, certainly supports the conclusion that MeHg exposures modulate the immune response in broader ways that elevate autoantibody production. The finding that elevated MeHg exposure was associated with decreased serum cytokine levels for the subset of the population with elevated ANA titers indicates a differential response to Hg in susceptible individuals. Although this study involved fish consumers in Brazil, the results are likely to be relevant in other populations, including those in the United States who consume large amounts of fish from the many watersheds affected by Hg deposition and listed by the U.S. Environmental Protection Agency for fish advisories. Thus, this research supports the importance of further research on the immunotoxic effects of MeHg exposures and the relevance of these effects for human health more generally.
